# A Negative-Stranded RNA Virus Infecting Citrus Trees: The Second Member of a New Genus Within the Order *Bunyavirales*

**DOI:** 10.3389/fmicb.2018.02340

**Published:** 2018-10-02

**Authors:** Beatriz Navarro, Stefania Zicca, Maria Minutolo, Maria Saponari, Daniela Alioto, Francesco Di Serio

**Affiliations:** ^1^Istituto per la Protezione Sostenibile delle Piante, Consiglio Nazionale delle Ricerche, Bari, Italy; ^2^Dipartimento di Agraria, Università degli Studi di Napoli Federico II, Portici, Italy

**Keywords:** bipartite genome, ambisense RNA, high-throughput sequencing, *Coguvirus*, survey, new virus

## Abstract

A new RNA virus has been identified from a sweet orange tree in southern Italy. This virus, tentatively named citrus virus A (CiVA), has a bipartite genome composed of (i) a negative-stranded (ns) RNA1, encoding the viral RNA-dependent RNA polymerase (RdRp), and (ii) an ambisense RNA2, coding for the putative movement protein (MP) and nucleocapsid protein (NP), with the two open reading frames separated by a long AU-rich intergenic region (IR) adopting a hairpin conformation. CiVA genomic RNAs and the encoded proteins resemble those of the recently discovered citrus concave gum-associated virus (CCGaV). This CCGaV, a nsRNA virus associated with the ancient citrus concave gum disease, has been proposed as the representative member of a new genus tentatively named *Coguvirus.* Molecular and phylogenetic analyses presented here support the classification of CiVA, and likely of other two recently described nsRNA viruses infecting plants, in this new genus. By showing that the evolutionary origin of the MP of all the putative coguviruses likely differs from that of their respective RdRp and NP, this study also provides evidence of a likely modular genome evolution for these viruses. Moreover, phylogenetic data support the proposal that, during the evolutionary history of nsRNA viruses, the plant-infecting viruses most likely emerged from an invertebrate-infecting ancestor several times as independent events. CiVA was identified in a field sweet orange tree not showing any obvious symptom and was graft-transmitted to sweet orange, grapefruit, rough lemon and Dweet tangor indicator plants that did not developed symptoms. The capacity of infecting citrus hosts of several species was also confirmed by a preliminary survey that identified orange, mandarin, clementine and lemon trees as natural hosts of CiVA in several fields of southern Italy, again without any obvious association with specific symptoms.

## Introduction

Based on high-throughput sequencing (HTS) many new negative-stranded (ns) RNA viruses, most of which infecting invertebrates (Li et al., 2015; Shi et al., 2016; Tokarz et al., 2018), have been recently reported. The phylogenetic relationships between these viruses prompted the International Committee on Taxonomy of Viruses (ICTV) to update the classification of the new order *Bunyavirales* to contain nine families (Maes et al., 2018). Recently, citrus concave gum-associated virus (CCGaV), a novel bunyavirus infecting citrus trees, was identified by HTS and shown to be associated with a severe and ancient citrus disease called concave gum (CG) (Navarro et al., 2018). CCGaV has peculiar characteristics, including a bi-segmented RNA genome not coding for any glycoprotein(s) and virions lacking an external envelope, that distinguish it from members of currently recognized taxa in the order *Bunyavirales.* However, CCGaV is phylogenetically related to species in the family *Phenuiviridae* and to several yet not-classified viruses identified mainly in arthropodes (Navarro et al., 2018). Consequently, establishment of a novel genus tentatively named *Coguvirus* in the order *Bunyavirales* has been recently proposed to allocate CCGaV (Proposals 2018.020P.N.v1. Coguvirus, submitted to ICTV on 28/01/2018, and 2018.017M.N.v1. Bunyavirales, submitted to ICTV on 21/07/2018). Here, we report a new nsRNA virus infecting citrus with molecular features and phylogenetic relationships with CCGaV, which strongly suggest that it is a prototype of a second species in the proposed genus *Coguvirus.* The results of preliminary field surveys in southern Italy documented the presence of this new virus, tentatively named citrus virus A (CiVA), in citrus trees of several species and in several citrus-growing areas.

## Materials and Methods

### RNA Isolation and HTS of cDNA Libraries

Leaf tissue, collected on 2016 from a non-symptomatic sweet orange tree (cv. Tarocco grafted on sour orange) grown in a commercial orchard in Southern Italy, was used for generating a cDNA library of small RNAs (16–30 nt). The library was generated from total nucleic acids (TNA) extracted with phenol-chloroform from leaves and sequenced (run 1 × 50) on an Illumina Genome HiScan Analyzer by Fasteris custom service (Fasteris, Switzerland) as reported previously (Di Serio et al., 2010).

### Assembling of Reads and Sequencing of the Full-Length Viral Genome

Raw reads generated by HTS were filtered for quality, trimmed and *de novo* assembled (k-mer 15–17) using the Velvet Software 1.2.08 (Zerbino and Birney, 2008). The resulting contigs were screened by BlastX on the NCBI databases for the homologous viral sequences and those sharing significant sequence identity with CCGaV were aligned along the two RNA components of this virus, thus generating a preliminary genome scaffold of a new virus. Such a scaffold was used to design specific primers to amplify by RT-PCR overlapping cDNAs covering the full viral genome sequence (**Supplementary Table S1**). Amplicons were gel-purified, cloned and sequenced by Sanger Sequencing Custom Service (Macrogen, Netherlands) according to standards protocols (Navarro et al., 2018). The 5′ and 3′ termini of both CiVA genomic RNAs were determined by 5′ and 3′ rapid amplification of cDNA ends (RACE) using the specific primers reported in the **Supplementary Table S1**.

### Sequence Analyses

RNA secondary structures were predicted by the Mfold Web Server (Zuker, 2003). ORF Finder at NCBI^1^ and PFAM^2^ database (Finn et al., 2016) were used to predict the potential ORFs and identify the conserved protein domains, respectively. Modeling, prediction and analysis of CiVA proteins were performed with Phyre2 web portal (Kelley et al., 2015). When indicated, PROMALS3D^3^ (Pei et al., 2008) was applied to generated multiple alignments of protein sequences and/or structures.

### Phylogenetic Analyses

Phylogenetic trees of genomic regions, including the core amino acid sequence of RNA-dependent RNA polymerase (RdRp) and the complete amino acid sequences of movement and nucleocapsid proteins (MP and NP, respectively), were built using MEGA7 (Kumar et al., 2016). Multiple alignments were generated by Cobalt^4^ or Clustal Omega (Sievers et al., 2011), then TrimAl (Capella-Gutiérrez et al., 2009) was used to remove poorly aligned regions, thus generating a final alignment that was used to infer the phylogenetic trees adopting the maximum-likelihood method (ML) (500 bootstrap replicates). The best-fit amino acid substitution models (LG + G for RdRp and NP, and WAG + G + F for MP phylogenetic trees) were determined using MEGA7.

### Bioassays, Detection, and Field Survey

Bioassays were performed by grafting bark tissues from the CiVA-infected tree to several indicator plants, including sweet orange [*Citrus sinensis* (L.) Osbeck, cv. Madame vinous], grapefruit (*C. paradisi* Macf.), rough lemon (*C. jambhiri* Lush), and Dweet tangor (*C. reticulata* Blanco x *C. sinensis*). At least three plants of each species were graft-inoculated. Mock-inoculations were performed using bark tissues harvested from a certified virus-free Tarocco plant, maintained in screen-house at University of Bari.

A conventional RT-PCR assay was specifically set up for the molecular detection of CiVA. The newly developed protocol was used to carry out, during 2016 and 2017, field surveys in several orchards located in Campania and Apulia region (Southern Italy) to assess the presence and incidence of CiVA infections. The primer pair Ka-1 (5′-TCCTGATGAAGTCTTAAGATCGC-3′) and Ka-3 (5′-TTGCAGTAGTGAGAAGGGAGT-3′) was designed to amplify a cDNA fragment of 620 nt of CiVA RNA2 (**Supplementary Table S1**). TNA (100 ng) were extracted and reverse-transcribed as reported previously (Navarro et al., 2017). An aliquot (2 μl) of the cDNA reaction was used for PCR amplification performed in a reaction volume of 25 μl containing 1.25 units of GoTaq polymerase (Promega, Madison, WI, United States) and a final concentration of 0.2 μM for each primer. After an initial denaturation at 94°C for 3 min, followed by 32 cycles at 94°C for 30 s, 55°C for 30 s, 72°C for 30 s and a final extension step at 72°C for 7 min, the reaction products were separated by electrophoresis on 1.4% agarose gels and visualized by UV light after ethidium bromide staining. In addition, all samples were also tested by RT-PCR for the presence of CCGaV, following the protocol described by Navarro et al. (2018).

## Results

### Identification of a Novel nsRNA Virus by Next Generation Sequencing

Assembly of the reads obtained through HTS of a cDNA library of small RNAs purified from leaves of a citrus tree, grown in southern Italy (Campania Region) and not showing any evident symptoms, generated a total of 7801 *de novo* contigs (K-mer 15 and 17). BLAST searches identified several contigs to be sequences of citrus exocortis viroid (CEVd), hop stunt viroid (HSVd), and citrus dwarfing viroid (CDVd). In addition, 35 contigs encoding deduced peptides with high amino acids (aa) sequence identity (38 to 100%) with the RdRp, the NP and the putative MP of CCGaV reported recently from citrus (Navarro et al., 2018), were also identified (**Supplementary Table S2**). These data suggested that a new virus, related to CCGaV, was present in the tested plant. The full-length genome of this virus, named citrus virus A (CiVA), was determined by Sanger sequencing of overlapping cDNA fragments and by 5′ and 3′ RACE.

### Genomic Organization of CiVA

**FIGURE 1:**
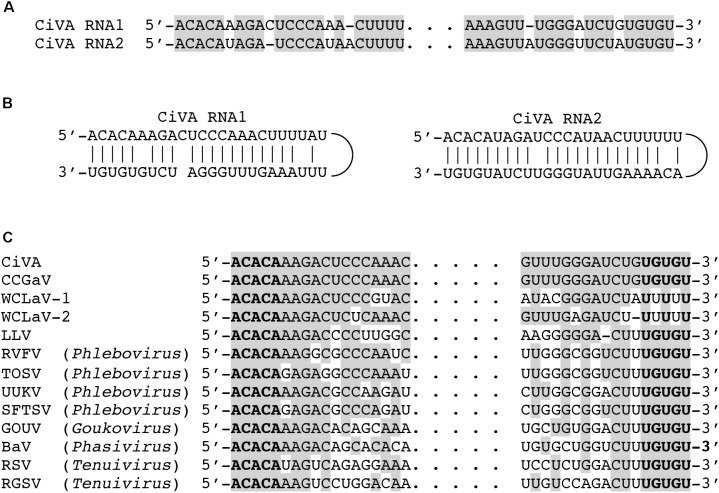
Terminal sequences of the citrus virus A (CiVA) genomic RNAs. **(A)** Alignment of 5′ (left) and 3′ (right) termini of RNA1 and RNA2 of CiVA; identical nucleotides (nt) are reported in gray. **(B)** Panhandle structures formed by the 5′ and 3′ termini of CiVA RNA1 and RNA2. **(C)** Alignment of CiVA RNA1 termini with those of other negative-stranded RNA (nsRNA) viruses; identical nucleotides are reported in gray, with the last conserved five nucleotides in bold. BaV, Badu phasivirus, (KT693187); CCGaV, citrus concave gum-associated virus (NC_035759); GOUV, Gouléako virus (HQ541738); LLV, Laurel Lake virus (KX774630); RGSV, rice grassy stunt virus (NC_002323); RSV, rice stripe virus (NC_003755); RVFV, Rift Valley fever virus (NC_014397); SFTSV, severe fever with thrombocytopenia syndrome virus (NC_018136); TOSV, Toscana virus (NC_006319); UUKV, Uukuniemi virus (NC_005214); WCLaV-1, watermelon crinkle leaf-associated virus 1 (KY781184); WCLaV-2, watermelon crinkle leaf-associated virus 2 (KY781187).

The CiVA genome is composed of two RNAs (RNA1 and RNA2) of 6691 and 2740 nucleotides (nt), respectively (GenBank accession numbers: MG764565 and MG764566). CiVA RNA1 and RNA2 share almost identical nucleotide sequences (up to 21 nt) at their 5′ and 3′ ends (**Figure 1A**). As expected for a nsRNA virus, the 5′ and 3′ termini of each genomic RNA are complementary (Bouloy, 2011) to each other, allowing the formation of a panhandle structure (**Figure 1B**). Interestingly, the 5′ and 3′ termini of both RNAs are identical to those of the CCGaV genomic RNAs (up to 18-nt at each terminus). Moreover, the five nt at both termini of CiVA and CCGaV genomic RNAs are identical to those of members of the family *Phenuiviridae* (genera *Phlebovirus, Phasivirus, Tenuivirus*, and *Goukovirus*) infecting animals and/or plants and to those of other bunyavirales-related viruses, such as the recently reported Laurel Lake virus (LLV) (Tokarz et al., 2018) infecting ticks (**Figure 1C**). These terminal sequences are also conserved, at least partially, in the 5′ end of the RNA1 of watermelon crinkle leaf-associated virus 1 (WCLaV-1) and watermelon crinkle leaf-associated virus 2 (WCLaV-2), which are two novel plant-infecting bunyavirales-related viruses reported in China (Xin et al., 2017; **Figure 1C**).

**FIGURE 2:**
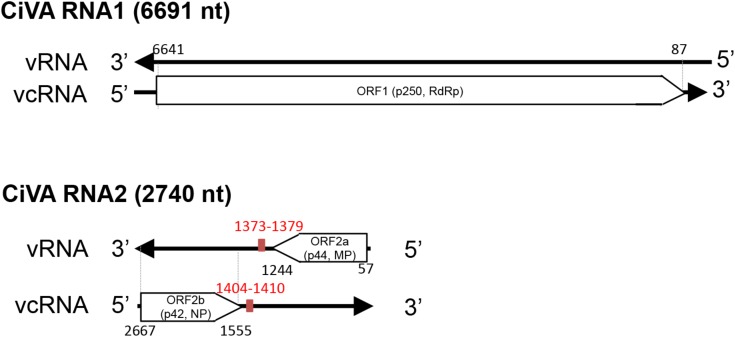
Schematic diagram of the citrus virus A (CiVA) genome. vRNA, viral RNA strand; vcRNA, viral RNA complementary strand; ORF, open reading frame; RdRp, RNA-dependent RNA polymerase; MP, movement protein; NP, nucleocapsid protein. In red, terminal transcription signals. Nucleotide positions with respect to the vRNA are reported.

CiVA RNA1 contains, in the viral complementary (vc) strand, a single open reading frame (ORF1) of 6582 nt (**Figure 2**), which starts at position 6639 and ends at position 89. ORF1 codes for a putative protein of 2184 aa with a predicted molecular mass of 250.5 kDa (p250). BlastP analysis of p250 identified the RdRp encoded by unclassified bunyavirales-related viruses from citrus (CCGaV), watermelon (WCLaV-1 and WCLaV-2) and ticks (LLV) as the closest related proteins, with an amino acid identity ranging from 77 to 33% (**Supplementary Table S3**). Accordingly, PFAM analysis showed that p250 contains the RdRp conserved domain of members of the order *Bunyavirales* between positions 556 and 1241 (Bunya_RdRp; E-value: 3.5e-46). Moreover, multiple alignments revealed in the CiVA p250, the typical six motifs (premotif A and motifs A–E) highly conserved in the RdRps of members of this taxon, showing the highest identity with CCGaV, WCLaV-1 and WCLaV-2, LLV and members of the genus *Phlebovirus* (**Supplementary Figure S1**). At the N-terminal region, p250 contains a conserved endonuclease domain (H_68_D_80_ PD_97-98_ ExG_109-111_ K_128_) probably involved in cap-snatching, a genome expression strategy of segmented nsRNA viruses of the order *Bunyavirales* and the families *Orthomyxoviridae* and *Arenaviridae* (Reguera et al., 2010; Decroly et al., 2012). Similar to CCGaV, WCLaV-1, WCLaV-2 and several orthomyxoviruses, an ExG motif instead of the ExK typical of most bunyaviruses has been present in this domain in CiVA p250. In addition, the amino acid doublet RY, also conserved in most bunyaviruses (Muller et al., 1994), was mapped at positions 599–600 of the same protein. Based on these data, the putative protein p250 is very likely the RdRp of CiVA.

**FIGURE 3:**
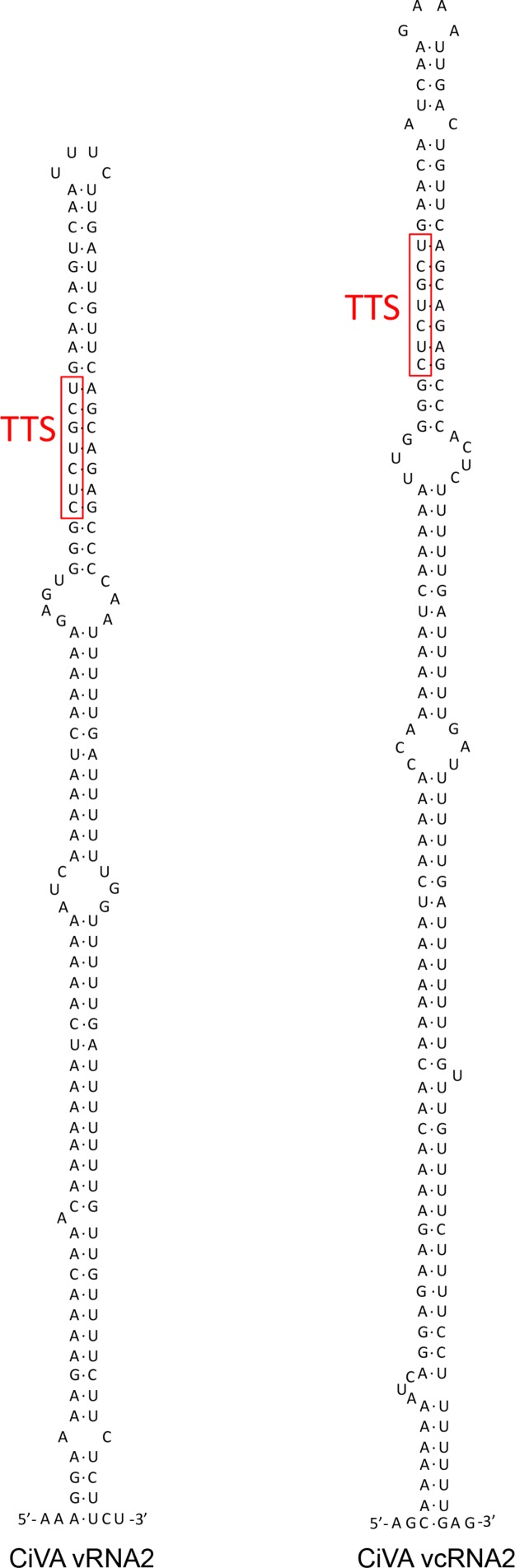
Stem loop and predicted transcription termination signal (TTS) motifs (in red) in the intergenic region (IR) of both RNA2 strands of citrus virus A.

CiVA RNA2 contains two ORFs (ORF2a and ORF2b) in opposite orientation (**Figure 2**), separated by an AU-rich intergenic region (IR) of 310 nt (AU content: 74.2%) that assumes compact conformations containing a long hairpin (**Figure 3**). A similar structural element has been previously reported in the viral (v) and in the vc strands of CCGaV RNA2. As proposed for other ambisense viruses of genera *Phlebovirus, Tenuivirus*, and *Orthotospovirus*, this highly structured region could act as a transcription termination signal (TTS) during genome transcription (Nguyen and Haenni, 2003). Interestingly, the hairpins formed by the CiVA IR in v and vc strands of RNA2 contain the motif CUCUGCU (**Figure 3**), also found at similar positions in the IR hairpins predicted for CCGaV RNA2 (Navarro et al., 2018) and previously proposed to be a TTS in negative-sense and ambisense RNAs of several phleboviruses (Albariño et al., 2007).

ORF2a, spanning from position 57 to 1244 of RNA2 (viral strand), codes for a putative protein of 395 aa with a molecular mass of 44.47 kDa (p44). Although PFAM search did not identify any conserved domain, BlastP analyses showed a significant identity of this protein with the putative MPs of CCGaV, WCLaV-1 and WCLaV-2 (E value 0.0, 2e-134 and 6e-116, respectively), supporting a similar functional role. Consistent with this view, the core domain of typical MPs of the 30K superfamily, formed by an α-helix followed by seven β-strands (Melcher, 2000), was identified in p44 by multiple alignment with PROMALS3D (**Supplementary Figure S2**). In this alignment, the nearly invariant aspartate residue (D) in the 30K specific motif (LxD/N_50-70_G) (Mushegian and Elena, 2015), and two conserved residues (a proline and an aliphatic amino acid at positions 155 and 202 in CiVA) (**Supplementary Figure S2**) crucial for the movement activity of the 30K MP of Ourmia melon virus (genus *Ourmiavirus*) (Margaria et al., 2016), were also identified in CiVA and in the related CCGaV, WLCaV-1, and WLCaV-2. Interestingly, CiVA p44 also has a significant identity with a putative protein encoded by RNA2 of the tick-infecting LLV (*E*-value 1e-33).

ORF2b, contained in the vc strand of RNA2 from nucleotide positions 2667 to 1555, encodes a putative NP of 370 aa and 42 kDa (p42) showing significant sequence identity with the putative NP of CCGaV, WCLaV-1, WCLaV-2, and LLV (**Supplementary Table S3**). In the region between positions 106–334 of p42, PFAM analysis mapped the domain Tenui-N (*E*-value 9.5e-16) conserved in the NP of viruses of the family *Phenuiviridae.* Moreover, Phyre2 analysis identified the N protein of Rift Valley fever virus (RVFV) as the best template for modeling (with 100% confidence) the tertiary structure of the p42 region between positions 108 to 334 (about 58% of the full-length protein). Noteworthy, similar results were obtained when Phyre2 analysis was performed with the putative NP encoded by CCGaV (Navarro et al., 2018) and WCLaV-1 and WCLaV-2 (data not shown), thus supporting close structural relationships between the proteins of these viruses. In addition, PROMALS3D multiple alignments of the CiVA NP with those of representative phleboviruses, tenuiviruses and phenui-like viruses (**Supplementary Figure S3**), showed α-helices consistent with those appearing in the Phyre2 predicted structure (data not shown), and provided further data. In particular, the following domains/motifs were identified in CiVA p42 and in the corresponding proteins of the phleboviruses and tenuiviruses included in the analyses: (i) two α-helices essential for NP protein oligomerization in RVFV, Uukuniemi virus (UUKV) and rice streak virus (RSV) (Katz et al., 2010; Ferron et al., 2011; Lu et al., 2017), (ii) a tyrosine stacking with the 5′ end of a bound RNA in RVFV and UUKM (Raymond et al., 2012; Mottram et al., 2017), (iii) three positively charged amino acids predicted to form a cleft involved in protein-RNA binding in RVFV and RSV (Lu et al., 2017), and (iv) the phenylalanine residue having an essential role in RFVF infectivity (Mottram et al., 2017; **Supplementary Figure S3**). In addition, PROMALS3D alignments also revealed that the NPs of CCGaV, WCLaV-1, WCLaV-2, and CiVA differ from those of all the other phenuiviruses due to the presence of a 100-aa segment at the N-terminal region forming three additional conserved α-helices (**Supplementary Figure S3**). Altogether these data, besides supporting that p42 is the CiVA NP, suggest that this virus is more closely related to CCGaV, WCLaV-1, and WCLaV-2 than to members of family *Phenuiviridae* and other bunyavirales.

### CiVA Is Phylogenetically Related to CCGaV and Other Bunyavirales-Related Viruses Infecting Plants

**FIGURE 4:**
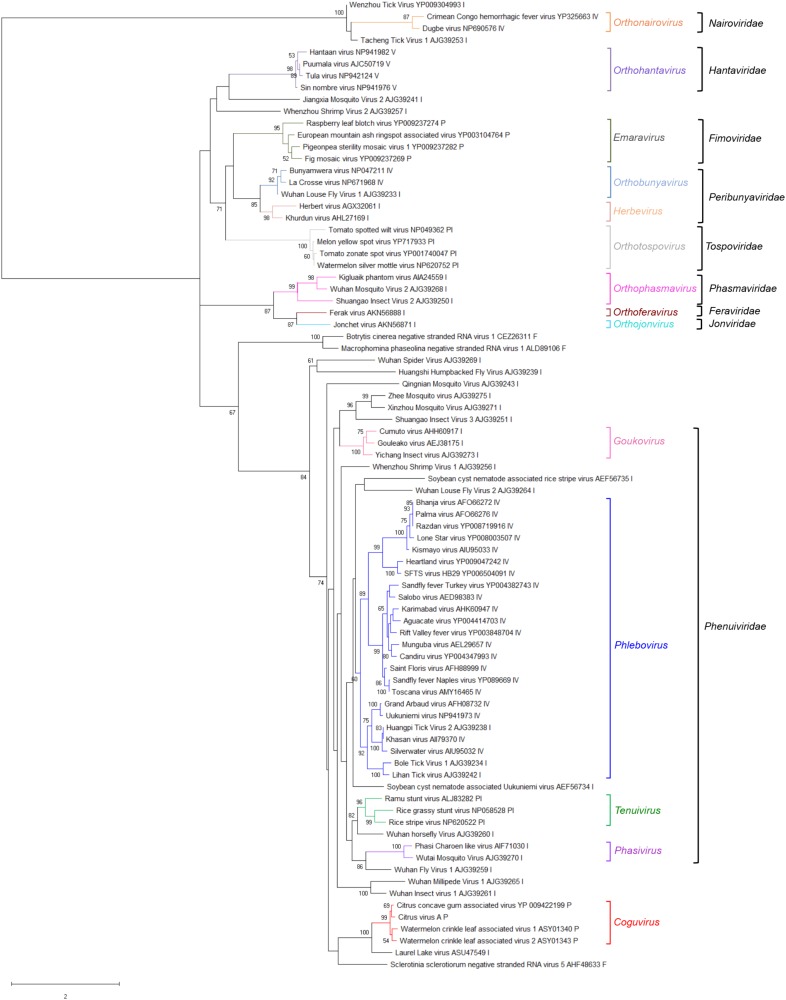
Phylogenetic tree of the RNA-dependent RNA polymerase (RdRp) conserved core domain of citrus virus A (CiVA) and 81 nsRNA viruses, representative of the order *Bunyavirale*s and several related unclassified viruses. Maximum likelihood method adopting the LG + G amino acid substitution model was used to infer the phylogenetic tree. Bootstrap probability values (500 replicates) above 50% are shown at branch nodes. Tree branches are proportional to the genetic distances, with the scale bar indicating substitutions per amino acid site. The names of the viruses, the accession numbers and the respective host organisms (invertebrate, I; vertebrate, V; invertebrate and vertebrate, IV; plant, P; plant and invertebrate, PI; fungus, F) are shown at the bra3nch tip. Recognized genera and families and the tentative genus *Coguvirus* are reported on the right.

**FIGURE 5:**
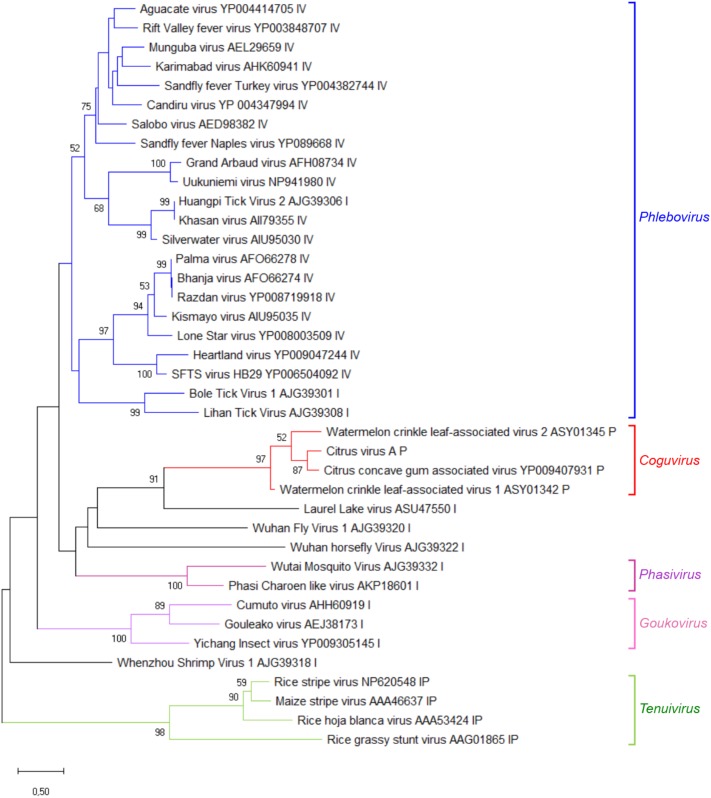
Phylogenetic tree generated using the nucleocapsid protein sequences of citrus virus A (CiVA) representative members of the family *Phenuiviridae* and several related unclassified bunyaviruses. Maximum likelihood method adopting the LG + G amino acid substitution model was used to infer the phylogenetic tree. Recognized genera and the tentative genus *Coguvirus* are reported on the right. Information on bootstrap values, distances and other symbols are reported in the legend of **Figure 4**.

A phylogenetic tree was inferred using the RdRp conserved core of 82 nsRNA viruses, including representative members of the order *Bunyavirales* and recently reported nsRNA bunyavirales-related viruses from plants and invertebrates (**Figure 4**). In such a tree, CiVA, CCGaV, WCLaV-1, and WCLaV-2 were clustered in a single clade, which, together with other non-classified nsRNA viruses mainly infecting arthropods, is nested at the base of a superclade also including all the other genera of the family *Phenuiviridae* (**Figure 4**). A cluster formed by CiVA, CCGaV, WCLaV-1, and WCLaV-2 was also observed in the phylogenetic tree inferred using the NP of CiVA and representative members of the family *Phenuiviridae*, thus supporting a close phylogenetic relationship between the four recently reported plant-infecting viruses (**Figure 5**). Interestingly, the phylogenetic trees based on RdRp and NP amino acid sequences showed that the tick-infecting LLV, whose RNA genomic terminal sequences are similar to those of CiVA and CCGaV (**Figure 1C**), is more closely related to these four viruses infecting plants than to other invertebrate-infecting nsRNA viruses.

**FIGURE 6:**
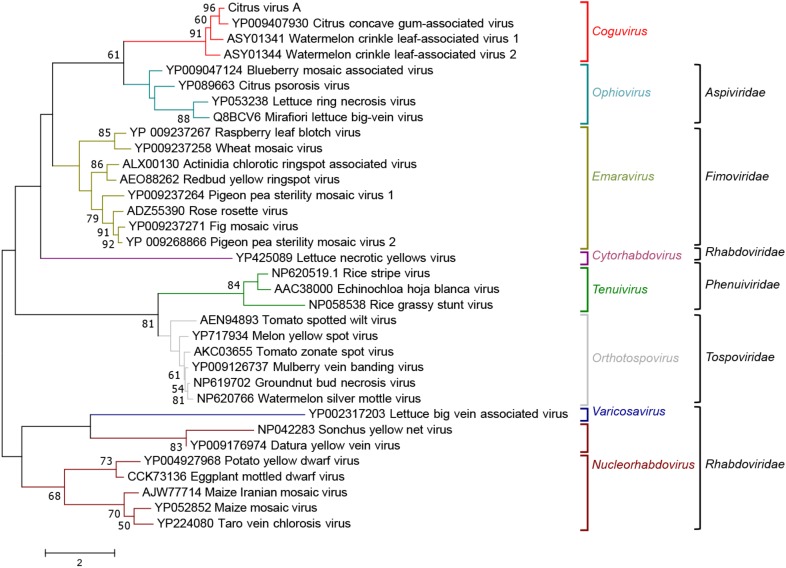
Maximum likelihood phylogenetic tree inferred with the putative movement protein sequences of citrus virus A (CiVA), other coguviruses and representative plant-infecting viruses of the genera *Emaravirus* (family *Fimoviridae*), *Ophiovirus* (family *Aspiviridae*), *Tenuivirus* (family *Phenuiviridae*), *Orthotospovirus* (family *Tospoviridae), Nucleorhabdovirus, Cytorhabdovirus*, and *Varicosavirus* (family *Rhabdoviridae*). The tree was generated using the WAG + G + F amino acid substitution model. Information on bootstrap values, distances and other symbols are reported in the legend of **Figure 4**.

When the phylogenetic tree was constructed using the MPs of representative nsRNA viruses infecting plants, CiVA, CCGaV, WLCaV-1, and WLCaV-2 were again grouped in a single clade (**Figure 6**) that, in this case, showed the closest relationships with members of the genus *Ophiovirus* (family *Aspiviridae*).

### Biological Characterization and Distribution of CiVA in Southern Italy

Following graft-inoculations of bark tissues from the CiVA-infected source onto indicator plants, the virus was successfully detected, using the newly developed RT-PCR assay, on all grafted indicator plants. In contrast, the mock-inoculated plants tested negative to CiVA infection. These results proved that CiVA is graft-transmissible to sweet orange [*Citrus sinensis* (L.) Osbeck, cv. Madame vinous], grapefruit *(C. paradisi* Macf.), rough lemon *(C. jambhiri* Lush) and Dweet tangor (*C. reticulata* Blanco x *C. sinensis*). Visual inspections carried out periodically up to 1 year after graft-inoculation, did not reveal the appearance of any leaf alteration or virus-induced symptom.

When samples from 71 citrus trees of different species, including sweet orange, clementine (*C. clementina* Hort. Ex Tan.) and mandarin (*C. reticulata* Blanco) trees from different surveyed orchards in Campania region (southern Italy) were assayed by RT-PCR, a total of 15 samples tested positive for CiVA infection, with at least one tree of each assayed host species being infected by the virus (**Table 1**). The trees tested in this survey were also assayed to investigate and confirm the association of CCGaV with CG disease (Navarro et al., 2018). Interestingly, in the present study CiVA was found in ten trees not showing CG symptoms and not infected by CCGaV. However, CiVA was also detected in two sweet orange and three clementine trees affected by CG and concurrently infected by CCGaV (**Table 1**). In addition, 22 lemon (*C. lemon* L.) trees from Apulia region (southern Italy) were also assayed, three of which tested positive to CiVA infection. Altogether these data, besides confirming the distribution of the virus in several citrus fields in southern Italy, extended the natural host range of CiVA, first identified in sweet orange, clementine, mandarin and lemon trees.

**Table 1:** Preliminary survey of citrus virus A (CiVA) in citrus trees of several species.

Citrus species	Tested	CiVA	CCGaV	CiVA and CCGaV
*Citrus sinensis*	50	10	18	2
*C. clementina*	18	4	10	3
*C. reticulata*	3	1	1	0
**Total**	**71**	**15**	**29**	**5**


## Discussion

In the present study, a novel nsRNA virus, named citrus virus A, has been identified by HTS and its full-length genome then completely sequenced by a classical molecular biology approach based on Sanger sequencing. CiVA is closely related to CCGaV, another nsRNA virus recently reported from citrus (Navarro et al., 2018). Both viruses, besides having bipartite genome composed of a negative sense RNA (RNA1) and an ambisense RNA (RNA2), share additional features that include: (i) identical sequences at 5′ and 3′ termini, (ii) a unique genome organization among plant-infecting nsRNA viruses, with the ambisense RNA2 bearing the MP and NP genes in opposite orientation, and (iii) a long AU-rich IR that adopts a hairpin conformation with a putative TTS located at similar position in either RNA2 strands. The amino acid sequence identity between the proteins encoded by CiVA and CCGaV was also the highest among the known nsRNA viruses. Due to the atypical bipartite negative/ambisense genome organization among nsRNA viruses and the unique arrangement of genes within the RNA2, CCGaV has been proposed to represent a novel species of a novel genus (*Coguvirus*) (Navarro et al., 2018). Given the structural and likely functional similarities at RNA and protein levels and the phylogenetic proximity between CCGaV and CiVA, the latter should be regarded as the member of a second species of this new genus.

Interestingly, in the phylogenetic trees inferred using RdRp, MP and NP the recently reported WCLaV-1 and WCLaV-2 always cluster together with CiVA and CCGaV. In contrast to CiVA and CCGaV, WCLaV-1 and WCLaV-2 have been reported to have tripartite genome composed exclusively of negative sense RNAs (Xin et al., 2017). However, the sequences of the RNA2 and RNA3 of the last two viruses available in GenBank (accessions KY781185 and KY781186 for WCLaV-1 RNA2 and RNA3, respectively; KY781188 and KY781189 for WCLaV-2 RNA2 and RNA3, respectively) have long U-rich 3′ terminal untranslated regions, with the five terminal nucleotides different from each other and from those conserved in all phenuiviruses. Moreover, in contrast to the nsRNA viruses with segmented genome, these termini are not complementary to those at the 5′ ends of the respective RNAs, suggesting that the sequences of RNA2 and RNA3 of WCLaV-1 and WCLaV-2 deposited in GenBank could lack the actual 3′ terminal nucleotides of the genomic RNAs. Taking this into consideration, and also that the genomic organization of CCGaV has been confirmed by Northern blot hybridization experiments showing the actual size of RNA2 and excluding the existence of an RNA3 (Navarro et al., 2018), the possibility that RNA2 and RNA3 of WCLaV-1 and WCLaV-2 may actually be two fragments of a single ambisense RNA with an organization similar to CCGaV and CiVA RNA2 (including a long intergenic AU-rich hairpin) should be considered. This alternative is also consistent with the phylogenetic analyses reported in the present study, which shows that, regardless of the viral protein considered, WCLaV-1, WCLaV-2, CiVA, and CCGaV consistently cluster together, thus supporting the classification of WCLaV-1 and WCLaV-2 as members of additional species in the genus *Coguvirus.*

When RdRp and NP encoded by CiVA were considered, our analyses highlighted close structural and phylogenetic relationships with the homologous proteins encoded by members of the recently created family *Phenuiviridae* (Maes et al., 2018). Similar results were obtained previously for CCGaV (Navarro et al., 2018) and WCLaV-1 and WCLaV-2 (Xin et al., 2017). However, whether the tentative genus *Coguviru*s should be classified in the family *Phenuiviridae* remains unclear. In fact, relevant differences in both CiVA and CCGaV with respect to phenuiviruses do exist, including: (i) the number of the genomic components (at least three in phenuiviruses), (ii) the absence in CiVA and CCGaV (and also in WCLV-1 and WCLaV-2) of genes coding for glycoproteins, which are instead encoded by phenuiviruses, (iii) the presence in the putative NP encoded by CiVA and CCGaV (and also by WCLaV-1 and WCLaV-2) of a N-terminal region with conserved structural elements absent in all the other phenuiviruses (**Supplementary Figure S3**), and (iv) a putative MP (in the four potential members of the tentative genus *Coguvirus*) phylogenetically related to members of the genus *Ophiovirus* (family *Aspiridae)* (García et al., 2017), rather than any plant-infecting phenuiviruses (included in the genus *Tenuivirus*) (Falk and Tsai, 1998). Some of these features are not restricted to CiVA and CCGaV, because the bipartite nature of their genome and the absence of glycoprotein(s) have also been reported in several other phenui-like viruses infecting invertebrates (Li et al., 2015; Shi et al., 2016; Tokarz et al., 2018). Relationships of members of the tentative genus *Coguvirus* with the other yet unclassified phenui-like viruses should be also taken into consideration while making decisions on whether such a genus should be classified in an extant or in a new family of the order *Bunyavirales.*

From an evolutionary point of view, the phylogenetic link of CiVA MP and all the other members of the tentative genus *Coguvirus* with the homologous proteins of ophioviruses (**Figure 6**), which are nsRNA viruses distantly related to bunyaviruses, is worth of note. This finding, showing that the MPs of all putative members of the genus *Coguvirus* likely have an evolutionary origin differing from those of their respective RdRp and NP, support a modular genome evolution (Koonin et al., 2015). Indeed, based on the Bayesian phylogenetic reconstruction of the ancestral host of CCGaV, it has been previously suggested that this virus most likely evolved from an invertebrate-infecting ancestor, while acquiring the MP from an ophiovirus-like virus independently later during evolution (Navarro et al., 2018). Based on phylogenetic trees inferred in the present study, a similar evolutionary scenario can be extended to CiVA, and to WCLaV-1 and WCLaV-2, possibly descending from the same or similar CCGaV ancestors. Consistent with this prediction is the relationship of CiVA, and all the other potential members of the tentative genus *Coguvirus*, with LLV (a virus infecting ticks) which, in the phylogenetic trees generated with RdRp and NP proteins, appears to be the closest relative among the known extant viruses (**Figures 4, 5**). A similar situation is also observed with the plant-infecting tenuiviruses and Wuhàn horsefly virus that infects insects, as shown in the RdRp phylogenetic tree and in previous studies (Li et al., 2015; Navarro et al., 2018), thus showing that close evolutionary links between plant- and invertebrate-infecting viruses are common among bunyavirales. Interestingly, the other taxa of plant-infecting nsRNA viruses are also directly nested with taxa including viruses infecting invertebrates, further supporting the view that emergence of plant-infecting viruses from an invertebrate-restricted ancestor likely happened through a series of multiple independent events (Navarro et al., 2018).

Infectivity of CiVA was ascertained by graft-transmission and its distribution in Southern Italy assessed by a preliminary survey that identified the virus in about 20% of the tested samples. Interestingly, this virus was neither associated with symptoms in the inoculated indicator plants nor with CG symptoms in the field, thus supporting that CiVA and CCGaV differ from a biological point of view. However, due to the limited extension of the survey, the possible association of CiVA with symptoms and, in particular, its relationship with some ancient citrus diseases of unknown origin, such as cristacortis and impietratura (Roistacher, 1991; Moreno et al., 2015), demand additional efforts and investigations. The available molecular tools, including the RT-PCR detection method reported in this study, may provide an answer to long-standing questions.

## Author Contributions

FDS, BN, DA, and MS conceived the project. FDS and BN supervised the project and wrote the manuscript. BN, SZ, MM, FDS, and DA performed the experiments and analyzed the data. All authors revised the manuscript.

## Conflict of Interest Statement

The authors declare that the research was conducted in the absence of any commercial or financial relationships that could be construed as a potential conflict of interest.

## Footnotes

1 http://www.ncbi.nlm.nih.gov/gorf/gorf.html

2 http://pfam.xfam.org/

3 http://prodata.swmed.edu/promals/promals.php

4 https://www.ncbi.nlm.nih.gov/tools/cobalt/re_cobalt.cgi
