# Supplementary material for: A Negative-Stranded RNA Virus Infecting Citrus Trees: The Second Member of a New Genus Within the Order Bunyavirales
Source: Front Microbiol. 2018 Oct 2;9:2340. doi: 10.3389/fmicb.2018.02340 (PMC6176071; doi:10.3389/fmicb.2018.02340)
Supplement: Supplementary file 1 [file Data_Sheet_1.PDF]

SUPPLEMENTARY MATERIAL

A negative-stranded RNA virus infecting citrus trees: the second member of a new genus within the order *Bunyavirales*

Beatriz Navarro, Stefania Zicca, Maria Minutolo, Maria Saponari, Daniela Alioto, Francesco Di Serio

Figure S1. Multiple alignment of RdRp conserved motifs of citrus virus A (CiVA) with other negative-stranded RNA viruses.

Figure S2. Multiple alignment with PROMALS3D of the putative movement protein (MP) encoded by citrus virus A (CiVA) and 30K MPs encoded by viruses representative of several genera.

Figure S3. Multiple alignment with PROMALS3D of the putative nucleocapsid protein (NP) encoded by citrus virus A (CiVA) and homologous proteins of representative phleboviruses, tenuiviruses and the phenui-like viruses more closely related with CiVA.

Table S1. Primers used in this study.

Table S2. Contigs coding for peptides with significant amino acid (aa) sequence identity with proteins of CCGaV.

Table S3. Identity between proteins encoded by CiVA and other related nsRNA viruses.

```
                          Premotif A                      Motif A                Motif B
                          870                        898  933             951  1026               1048
CiVA                      K-AQHGGDREIHVLEIKMRVLQFFCESMSK  QFFTMGKSADATKWCQRND  KMFIRSGMMQGILHYTSSFTHTL
CCGaV                     K-SQHGGDREIHVLEVKMRVLQYFVESMSK  QFFTMGKSADATKWCQRND  KMFIKSGMMQGILHYTSSFTHAM
WCLaV-1                   K-AQHGGDREIHVLDITMRIIQYFAECISR  NFFTMGKSADATKWCQRND  KIMIRSGMMQGILHYTSSFTHSV
WCLaV-2                   K-AQHGGDREIHVLEIKMRIIQFFTESMSK  QFFTLGKSADATKWCQRND  KMMIRSGMMQGILHYTSSITHAV
LLV                       K-SQHGGEREIHVLEFKARIVQYFVELIAR  NFTTISKSADATTWCQFHH  LIEVISGMFQGILHTTSSLYHTM
TOSV   (Phlebovirus)      K-QQHGGLREIYVMGADERIVQSVIEAIAR  SVWTCATSDDARKWNQGHY  YLKTSTGMMQGILHFTSSLLHSL
UUKV   (Phlebovirus)      K-PQHGGLREIYVLGFEERVVQLVIETIAR  HHETVATSDDAAKWNQCHH  FVQTETGMMQGILHYTSSLLHTL
HuTV   (Phlebovirus)      KKPQHGGLREIYVLGFEERVVQLVLETIAR  QHQTVGTSDDARTWNQGHH  FIQTETGMMQGILHFTSSLFHTI
KHAV   (Phlebovirus)      KKPQHGGLREIYVLGFEERVVQLVLETIAR  QHQTVGTSDDARTWNQGHH  YIQTETGMMQGILHFTSSLFHTI
BHAV   (Phlebovirus)      K-AQHGGLREIYVLRLEERMIQFGIELLAR  TMITLCSSNDARTWNQGHY  YILTETGMMQGILHLTSSLYHAA
SFTSV  (Phlebovirus)      K-NQHGGLREIYVMDANARLVQFGVETMAR  GSININSSNDAKKWNQGHY  YIKTETGMMQGILHFTSSLLHSC
RSV    (Tenuivirus)       K-NQHGGLREIYVLNIFERIMQKTVEDFSR  EYMTISTSDDASKWNQGHY  YIETETGMMQGILHYTSSLFHAI
HTNV   (Orthohantavirus)  KYQRTEADRGFFITTLPTRCRLEIIEDYYD  KRKLMYVSADATKWSPGDN  HGEVKGNWLQGNLNKCSSLFGVA
BUNV   (Orthobunyavirus)  KGQKTAKDREIFVGEFEAKMCMYVVERISK  ALK-LEINADMSKWSAQDV  YVQIKRNWLQGNFNYISSYVHSC
EMARaV(Emaravirus)        KDQRTTDDREIYTGNAQVRLCLYPLEMTFK  KTEIYSVSSDASKWSARDL  WFNVRSNWLQGNLNMTSSFVHHC
TSWV   (Orthotospovirus)  KMQRTKTDREIYLMSMKVKMMLYFIEHTFK  KSRLAFLSADQSKWSASGL  TYPVSMNWLQGNLNYLSSVYHSC
DUGV   (Orthonairovirus)  K-AQLGGSRDLLVQETGTKVIHATTEMFSR  FFKTVCISGDNTKWGPIHC  AMNSYNHMGQGIHHATSSLLTSM
                          *  :    * :       :      *           . *  .*           **  :  **

                          Motif C          Motif D         Motif E
                          1067       1081  1110      1123  1127    1138
CiVA                      HCNITVVQGSDDSAQ  YFSIYTSRAKSSIG  LIEYNSEWMVRN
CCGaV                     LCNITVVQGSDDSGQ  YFSIYTSRAKSSIG  LIEYNSEWMVRS
WCLaV-1                   ESYITVVQGSDDSAE  QFSIYTSRAKSSIG  LIEYNSEWMIRS
WCLaV-2                   DSYITVVQGSDDSAE  QFSIYTSRAKSAIG  LVEYNSEWMIRS
LLV                       KVHITVCQGSDDSGC  YLSVFCNEAKSSIG  LIEYNSEWHVRH
TOSV   (Phlebovirus)      KVVCDMMQGSDDSSM  YIGIYPS-EKSTPN  VMEYNSEFFFHS
UUKV   (Phlebovirus)      DVLVDVLQSSDDSGM  YLGIYSS-VKSTNN  LLEFNSEFFFHI
HuTV   (Phlebovirus)      NPHVDVLQSSDDSGM  LIGIYPS-VKSTSN  VLEFNSEFFFHT
KHAV   (Phlebovirus)      NPHVDVLQSSDDSGM  LIGIYPS-IKSTSN  VLEFNSEFFFHT
BHAV   (Phlebovirus)      VILIDVIEGSDDSAI  YAGIYMS-PKSTIG  VCEYNSEFEFRF
SFTSV  (Phlebovirus)      SGVVDVIEGSDDSAI  LFGIYSS-EKSTVN  CVEYNSEFHFRH
RSV    (Tenuivirus)       SCIVNNMESSDDSSF  YLGIYKS-PKSTTQ  VMEFNSEFFFSG
HTNV   (Orthohantavirus)  DCFFEFAHHSDDALF  GSIKISP-KKTTVS  NAEFLSTF-FEG
BUNV   (Orthobunyavirus)  DCLINSMVHSDDNQT  GCQA-NM-KKTYIT  CKEFVSLFNLHG
EMARaV(Emaravirus)        EAVMTSMVHSDDSTY  KHCITLN-EKKTYI  YKEFLSTTIVSN
TSWV   (Orthotospovirus)  DFQTRWIVHSDDNAT  FCITLNP-KKSYAS  EVEFISERISKW
DUGV   (Orthonairovirus)  TVNVDHAGSSDDYAK  VRRCCQM--KDSAK  FLEFYSEFMMGN
                                   ***              *           *
```

Figure S1. Multiple alignment of RdRp conserved motifs of citrus virus A (CiVA) with other negative-stranded RNA viruses. Positions in the CiVA RdRp are reported. In red, small and hydrophobic amino acids (aa), including aromatic ones (AVFPMILW); in blue, acidic aa (DE); in magenta, basic aa (RK); in green, polar (STNQ), aromatic (YH), cystein (C) and glycine (G). BHAV, Bhanja virus (AFO66272); BUNV, Bunyamwera virus (NP_047211); CCGaV, concave gum-associated virus (YP_009422199); DUGV, Dugbe virus (NP_690576); EMARaV, European mountain ash ringspot-associated virus (YP_003104764); HTNV, Hantaan virus (NP_941982); HuTV, Huángpí tick virus 2 (AJG39238); KHAV, Khasan virus (AII79370); LLV, Laurel Lake virus (ASU47549); RSV, rice stripe virus (NP_620522); SFTSV, severe fever with thrombocytopenia syndrome virus (YP_006504091); TOSV, Toscana virus (P37800); TSWV, tomato spotted wilt virus (NP_049362); UUKV, Uukuniemi virus (NP_941973); WCLaV-1, watermelon crinkle leaf associated virus 1 (ASY01340.1); WCLaV-2, watermelon crinkle leaf associated virus 2 (ASY01343).

```
        ID            Genus           aa position
CiVA                                   125 -EDTEQFNLS---------LAVPKK-------------------GKPYIR
CCGaV   YP_009407930                   122 -DDTEQFNLS---------LAVPKK-------------------GKQYVR
WCLaV-1 ASY01341                       123 -EQSEQLNLS---------MAIDKK------------------GKKEYIR
WCLaV-2 ASY01344                       121 -EDCKQFNLS---------EVVNMK------------------KGKPYIR
TSWV    AEK06236      Tospovirus       111 --ANKVIKIC------------------------PTWD-----SRKQYMM
RCNMV   NP_620546     Dianthovirus      33 --GVSEAPLI------PASMMSKIT--DYA---KTTAK-----GNSVALN
SBWMV   NP_049337     Furovirus         44 --RYVEKALT------QPGVVTKMQ--DAW---TNWTKTNKEEGTPYNMS
CMoV    YP_002302261  Umbravirus        42 --TLTHTPLY------PPRRQLTLS--RFF---SGRWR---TKKTGGMLF
CNSV    NP_733974     Nepovirus B      432 -----VLNRP------EYNALNKLA--------EAGWK---------EAK
OuMV    YP_002019758  Ourmiavirus       57 --VEDPIALI------PHGIWSIFK--SKL---AQMR------CPKGYIT
PoLV    YP_009032637  Aureovirus        30 --TKAILPLA------PISQFSKWK-----------------IPKQGFYA
CPsV    YP_089663     Ophiovirus        84 --HKKKLKLG------TLKSITDKL--------RKLGG----ESSQPFIQ
CPMV    NP_734000     Comovirus        204 --TVLDIPVT------KDKTLAMAS--------DFIRK---ENLKTSAIH
CiLV-C  YP_654542     Cilevirus         47 --RIGVGFLT------PNDMISRLV--GFI---NRKAEDAGVRSVESFRQ
CRLV    YP_081449     Cheravirus       109 --DPYVAVYTPL-PHITEEQARKLM-----------------EKGFSNSS
GFLV    NP_733995     Nepovirus A      118 --NTLEILYNP----VSEEEMEEYR-----------------DRGMSAVV
RSV     NP_620519     Tenuivirus        88 ----ATFNIL------VDPYWFHQPLTHYPF-FRVTHY-----PFFRVET
ACLSV   NP_040552     Tricovirus        62 --RLASIPVI------PSSEVQAVL--------RKR-------ESTNYVH
SDV     NP_733976     Sadwavirus        53 --QQQDIAIP------LSSRFG----------------------NHQSLH
PYMoV   YP_008567619  Badnavirus       100 --NQVDRTFI------QEESHKKLV-----------------KSGIKFIH
RRV     YP_004327592  Emaravirus        63 --KTQLLPIS------VYNEMYKFY--------MHL--------KQPMTR
TMV     NP_597748     Tobamovirus       36 CSKVDKIMVHENESLSEVNLLKGVK-----------------LIDSGYCL
CLBV    NP_624334     Citrivirus        55 --IKLQANIL------TADRLQSIR--------NAKVN----GKEAAYLH
PYFV    NP_734443     Sequivirus       115 ---RAEITPT------AASKLDKTL--------EAHRA------KFNYMA
RBDV    NP_620466     Idaeovirus       138 --GKNEFSLV------EASVFDKLI--------RKD---------DSPIH
TBSV    NP_062900     Tombusvirus       32 --EDQEVPLL------PSNFLTKVR----------------VGLSGGYIT
ToTV    YP_001040018  Torradovirus      66 ---KKAMELF------DPEDVHNIT--SLWSSFKKTFTS-SRDHGNLFFH
CaMV    NP_056724     Caulimovirus      97 --GKVYLPLI------TKEEINKRL--SSL---KPEVR-----KTMSMVH
CMV     NP_040776     Cucumovirus       52 --AISVRPLV------PEVTHGRIA--SFF---KSGYDV-GELCSKGYMS
AMV     NP_041194     Alfamovirus       60 --APVVLSLV------SKEKKSILN--RML---PKI-------GQRMYVH
LNYV    YP_425089     Cytorhabdovirus   53 --IMRQVPLF------DKEDLDAMK--------SDT-------TSNKYLH
AcVB    YP_004935360  Vitivirus         78 --VDQNIDLV------DEKTIDGLN-----------------EEKQPYLH
CaLCuV  NP_624353     Begomovirus       38 ---LTEILLQ------FPSTAAQFT--ARL--------------NRSCMK
Consensus_ss:                                   eeee        hhhhhhhh                      eee

                                                     *                     #
CiVA                                       LASVTAYYCPLVSS-----FSEFTKAGMSLHDSRLS--------SDTSVQ
CCGaV   YP_009407930                       LSSVMAYYCPLVSS-----FSEFTKVSISLHDSRLL--------SKTCVQ
WCLaV-1 ASY01341                           LASVFGIYVPLVSV-----FTKYTSVIVSLHDSRMC--------DETTIQ
WCLaV-2 ASY01344                           LASLIGIYTPLVSS-----FSDFSRVCVDLTDIRKL--------TDQSVQ
TSWV    AEK06236      Tospovirus           ISRIVIWVCPTIPN-------PTGKLVVALVDPNMPSE-------KQVIL
RCNMV   NP_620546     Dianthovirus         YTHVVLSLAPTIGV------AIPGHVTVELINPNVEGP-----FQVMSGQ
SBWMV   NP_049337     Furovirus            YSCVLLNVIPTVPM------GYAGTVEVSLLDSGLSPL-----ENVIPDQ
CMoV    YP_002302261  Umbravirus           IEKLVIVFVPHVPD------TSPGQAVIWVHDTALPGL-----EPIGTGQ
CNSV    NP_733974     Nepovirus B          SVVLNLHIRSYLPQ------QMNAYAFCVIMWGHSSDA------QEAALS
OuMV    YP_002019758  Ourmiavirus          YDKVILSWKPHVAT-------GLARGQIAVVDTRVNHTSIEDLMHKALWK
PoLV    YP_009032637  Aureovirus           PIDVKFVLTPHISE------RAQVRGVVKLVDSRDLSP------SRELYR
CPsV    YP_089663     Ophiovirus           FYKVQCMYIPLFSR----VDGDNGEITVSLIDDGKEAA-----GQDPIIQ
CPMV    NP_734000     Comovirus            IGAIEIIIQSFASP------ESDLMGGFLLVDSLHTDT------ANAIRS
CiLV-C  YP_654542     Cilevirus            ISDVVLIIVPQIA--------LPAELSLKLVDSANI--------LEAVNQ
CRLV    YP_081449     Cheravirus           NVALDMAVQSHVGQ------GTPLLAMCGIMDSRTDDP------NEALQV
GFLV    NP_733995     Nepovirus A          IDALEIAINPFGMP------GNPTDLTVVATYGHERDM------TRAFIG
RSV     NP_620519     Tenuivirus           FAMVWIGIKG----------RASGITTLRIIDKSYVNP------SDQVEV
ACLSV   NP_040552     Tricovirus           WGALSISIDALFRK------NAGVSGWCYVYDNRWETF------EQAMLQ
SDV     NP_733976     Sadwavirus           VGELEIAVQSSVLT------GVDTAMAIMVSDASHDRL------EEGFLS
PYMoV   YP_008567619  Badnavirus           LGVLQVRLQILHRK------EEGTLALVVFRDNRWKG-------DKGIIA
RRV     YP_004327592  Emaravirus           IASVALFWTPTSK-------RFNEMATLILMDERFRDDGI-KQGKKELLA
TMV     NP_597748     Tobamovirus          A-GLVVTGEWNLPD------NCRGGVSVCLVDKRMERA------DEATLG
CLBV    NP_624334     Citrivirus           LGFVPIAIRSLLPS-----GNEQIWGRCALVDTSRTRA------ETAVID
PYFV    NP_734443     Sequivirus           IDSIRVAVTSLMHQ------GDSRECIMYLCDRRFKDP------LLGAIA
RBDV    NP_620466     Idaeovirus           LNRLLIAVLPAVGK------GTPGTARIKIRDARLDDG------YGELFS
TBSV    NP_062900     Tombusvirus          MRRIRIKIIPLVSR------KAGVSGKLYLRDISDTT-------GRKLHC
ToTV    YP_001040018  Torradovirus         LYGVMFFMVPHVHG-------GEGSVKISLCSSNDP--------TNPVLQ
CaMV    NP_056724     Caulimovirus         LGAVKILLKAQFRN------GIDTPIKIALIDDRINSR------RDCLLG
CMV     NP_040776     Cucumovirus          VPQVLCAVTRTVST------DAEGSLRIYLADLGDKEL------SPIDGQ
AMV     NP_041194     Alfamovirus          HSAIYLLYMPNILK------SSSGSITLKLFNEATGEL---------VDV
LNYV    YP_425089     Cytorhabdovirus      IGCITVSIEPLLHQRYMKNFGKTIAGNCAIIDSTFRKV------DQSIIS
AcVB    YP_004935360  Vitivirus            LGCVAIAVIPHGR-------AMKGTVQIKVEDQRFKEG-------HGTVC
CaLCuV  NP_624353     Begomovirus          IDHCVIEYRQQVPI------NATGSVIVEIHDKRMTD-------DESLQA
Consensus_ss:                              eeeeeeeee               eeeeeee                eee
```

```
                                                                         *
CiVA                                 SVTFNSNI--------------TQKLELSLDYCIPRSSA-----------
CCGaV  YP_009407930                  SADFNSNI--------------TQKVELSLDYCIPRTSC-----------
WCLaV-1 ASY01341                     SVKFNSNI--------------PQKFELSLDYCIPRSEA-----------
WCLaV-2 ASY01344                     VVRFNSNV--------------PEKFELSLDYCIPRESA-----------
TSWV   AEK06236     Tospovirus       KGQGTITD--------------PICFVFYLNWSIPKMNNT---------P
RCNMV  NP_620546    Dianthovirus     TLSWSPGAG------------KPCLMIFSVHHQLNSDHE-----------
SBWMV  NP_049337    Furovirus        TQMMELGKG-------------PNVMCFFMHYSIPLNDKG---------R
CMoV   YP_002302261 Umbravirus       KVCIPLSSG-------------PRLVAFYPNYSIPLSDSAM-------NA
CNSV   NP_733974    Nepovirus B      GSYVYLGDG-------------EATMLQLPLLCEYVGHNLQD----FEAY
OuMV   YP_002019758 Ourmiavirus      TAPVDLGCT-------------YTIQGT-VPYCLPFHPKEGGDVKSDLES
PoLV   YP_009032637 Aureovirus       SKEFNIGHG-------------LVIEGSQLPFCLPVGD------------
CPsV   YP_089663    Ophiovirus       SITFDASQ--------------MAMVELSMNFFVEKKDM-----------
CPMV   NP_734000    Comovirus        IFVAPMRGG------------RPVRVVTFPNTLAPVSCD----------L
CiLV-C YP_654542    Cilevirus        EVTVNSTGG-------------PCVVVMNCAHSIPNEDRTHVN---GSEV
CRLV   YP_081449    Cheravirus       AGYFDLGRD-------------RCDLISLPLINFPLNKEDF------DDY
GFLV   NP_733995    Nepovirus A      SASTFLGNG-------------LARAIFFPGLQYSQEEPR---------R
RSV    NP_620519    Tenuivirus       EVRYPISKN-------------FAVLGSLAN-FLALEDK-----------
ACLSV  NP_040552    Tricovirus       KFHFNLDSG-------------SATLVTSPNFPVSLDDPG---------L
SDV    NP_733976    Sadwavirus       LTILRLGAG-------------WMRHTIPIGITVFPTDPL---------V
PYMoV  YP_008567619 Badnavirus       TMEVDLTKG-------------CQMVYIIPDIMMTVNDF-----------
RRV    YP_004327592 Emaravirus       QGKLNLDLMGTVITAVPFDPNFQQFVTGSLDFATDTKDI-----------
TMV    NP_597748    Tobamovirus      SYYTAAAKK-------------RFQFKVVPNYAITTQDAM----------
CLBV   NP_624334    Citrivirus       EFEFKFTKKQP---------FASKLLTINAAVDINCKVSV----------
PYFV   NP_734443    Sequivirus       LIGFTLPGMQTH------VYKTGRMMAFSRKEAIAAD-------------
RBDV   NP_620466    Idaeovirus       -SENRVDSG--------------VIYCINVGYSVPKSEI-----------
TBSV   NP_062900    Tombusvirus      TESLDLGRE-------------IRLTMQHLDFSVSTRSD-----------
ToTV   YP_001040018 Torradovirus     EKVLYFSGG-------------AQAVLMSPTITLPFVKR-----------
CaMV   NP_056724    Caulimovirus     AAKGNLAYG-------------KFMFTVYPKFGISLNTQR---------L
CMV    NP_040776    Cucumovirus      CVSLHNHDL-------------PALVSFQPTYDCPMETVG--------NR
AMV    NP_041194    Alfamovirus      DTDHDATQ--------------ACIFAGRYPRSILAKDAA---------K
LNYV   YP_425089    Cytorhabdovirus  LHKYDLSRG-------------RADYVSYPNHCLSLTDPM---------I
AcVB   YP_004935360 Vitivirus        SFKCDLKDA-------------LSAYASFPGYFVSTTDVK---------N
CaLCuV NP_624353    Begomovirus      SWTFPLRCN-------------IDLHYFSSSFFSLKDPI-----------
 Consensus_ss:                       eeeee                  eeeee    eee


CiVA                                 -SKITLNIAREQKFL-KEGEEWGAVQLLIKL---
CCGaV  YP_009407930                  -SKITLNIAREQKFL-QEGEEWATVQLLIRL---
WCLaV-1 ASY01341                     -SFISLNISREQTFM-REDSQWAVMQLTLKL---
WCLaV-2 ASY01344                     -DKIILNVALEQAFL-IRGEQWGTLQMMLII---
TSWV   AEK06236     Tospovirus       ENCCQLHLMCSQEY--KKGVSFGSVMYSW-----
RCNMV  NP_620546    Dianthovirus     --PFRVRITNTGI---PTKKSYARCHAYW-----
SBWMV  NP_049337    Furovirus        AVKLAFKIDAEMA---SKGMSVMNVYSYW-----
CMoV   YP_002302261 Umbravirus       PRCFSLVTQLEGVRL-EQGASAFSLYSMW-----
CNSV   NP_733974    Nepovirus B      KRSLVLSTVFPEFSGIADGKAMFGITSIEF----
OuMV   YP_002019758 Ourmiavirus      QNPIRGIVYITDSRY-------------------
PoLV   YP_009032637 Aureovirus       -YPLQFEVTVLQSQF-RETANLYSTSVEW-----
CPsV   YP_089663    Ophiovirus       -DFIGIHVSAENVPV--QDRAYGSINLAFF----
CPMV   NP_734000    Comovirus        NNRFKLICSLPNCDI-VQGSQVAEVSVNV-----
CiLV-C YP_654542    Cilevirus        HRRLGIQYQVDCDNI-SGRVTTFSITALW-----
CRLV   YP_081449    Cheravirus       MRGLYLCTMFHNVRGFQNNKALCSYSA-------
GFLV   NP_733995    Nepovirus A      ESIIRLYVASTNATV-DTDSVLAAISVGT-----
RSV    NP_620519    Tenuivirus       -HNLQVSVSVDDSSV---QNCVISRTLWFW----
ACLSV  NP_040552    Tricovirus       SNSISVAVMFENLNF-KFESYPISVRVGN-----
SDV    NP_733976    Sadwavirus       DRFLRLSVLTGGSPM-ADGRQVARLHYGL-----
PYMoV  YP_008567619 Badnavirus       YHNIHISVLTRGYEGWQHGEANLLVTRGM-----
RRV    YP_004327592 Emaravirus       -NKIKFYISFPDTRM-------------------
TMV    NP_597748    Tobamovirus      KNVWQVLVNIRNVKM-SAGFCPLSLEFVSVCIVY
CLBV   NP_624334    Citrivirus       -GSIQVLLELHGVDL-REERSVAAII--------
PYFV   NP_734443    Sequivirus       --RLQLYLYVKGAKLERTQNTPITVNVRT-----
RBDV   NP_620466    Idaeovirus       --DYKINIDFAGVPI-KDGKSPIWVKAAF-----
TBSV   NP_062900    Tombusvirus      -VPIVFGFEELVSPF-LEGRELFSISVRW-----
ToTV   YP_001040018 Torradovirus     GPMFYYTMECLGTRA----QIPCSVVAIW-----
CaMV   NP_056724    Caulimovirus     NQTLSLIHDFENKNLMNKGDKVMTITYVV-----
CMV    NP_040776    Cucumovirus      KRCFAVVIERHGYIQ--YTGTTASVCSNW-----
AMV    NP_041194    Alfamovirus      GHDLKLVVHAVASTN--ANSAVGVLYPIW-----
LNYV   YP_425089    Cytorhabdovirus  QKRLSVLLGIKGIDV-EPGVELFSICIGY-----
AcVB   YP_004935360 Vitivirus        GYALNLKVKAEGMCM-VEGVHPLSIQMHC-----
CaLCuV NP_624353    Begomovirus      --PWKLYYRVSDTNV-HQRTHFAKFKGKL-----
Consensus_ss:                           eeeeeeeee         eeeeeee
```

Figure S2. Multiple alignment with PROMALS3D of the putative movement protein (MP) encoded by citrus virus A (CiVA) and 30K MPs encoded by viruses representative of several genera. The virus name, GenBank identifier, virus genus and distance in amino acids from the N- terminus of the protein are shown on the left-hand side of each sequence. The distance in amino acids from the N ‐ terminus of the protein are shown on the left ‐ hand side of each sequence. Consensus secondary structure elements, predicted by PROMALS3D, are reported at the bottom, with the strands and helices indicated by e and h, respectively. Amino acids that fold in strands and helices and are in blue and red, respectively. The symbols # and * on the top indicate the nearly invariant aspartic amino acid residue (D, in bold, position 172) (Mushegian and Elena, 2015), and a proline and an aliphatic aa (in bold, at positions 155 and 202 in CiVA, respectively) shown to be crucial for movement activity of the 30K MP of OuMV (Margaria et al., 2016), respectively.

```
CiVA          1  MALHQNPKE--QSKFDALFSQATSHGEEHYREFLTKCSKNLKERIKREVNARK--MTSGGDFLESFEQAE   66
WCLaV_1       1  MD---HTN----AELIALINEKSSLGQAEFNKWFESLSKNNKSYVQKEMKARK--LAKAMTPSA-VEATE   60
WCLaV_2       1  MSSSSRTNNTLREQMKALKNKPES----EQSAWLEGLNEQEMRFVETQMKTTKGIKLAQTTAASSSETSE   66
CCGaV         1  MADKINNPS-TQAEFEAIYHDATSQDQAYFVAYFKRCSKALQERLKKEISARK--LIGAG----------   57
LLV           1  M---------------------------------------------------------------------    1
RSV           1  MGT----------------NKPA--TLADLQKAI------------------------------------   16
RGSV          1  MGK-----------------------VQFGDGHW---ANN------------------------------   14
MSV           1  MAT----------------NKPA--NLNDLQKAI------------------------------------   16
RHBV          1  MTM---------------------SVADVQTEI------------------------------------   12
EHBV          1  ------------------------MSVADIQTEI------------------------------------   10
UHBV          1  MAM---------------------SVADVQTEI------------------------------------   12
IWSV          1  MSM---------------------SVAEIQAEI------------------------------------   12
RVFV          1  M---------------------------------------------------------------------    1
SFTSV         1  MS--------------------------------------------------------------------    2
SFNV          1  MSD-------------------------------------------------------------------    3
UUKV          1  MAM-------------------------------------------------------------------    3
TOSV          1  MSD-------------------------------------------------------------------    3
Consensus_ss:          hhhhhhhhhhhhh       hhhhhhhhhhhhhhhhhhhhhhhhhhh          hhhhhhh

CiVA         67  DKSGKLDNKEGELEEDEHEDEEQEVTEGSGTKKDIVVTDAQLAAMWTEIDN----IDVSTFDRE---STK  129
WCLaV_1      61  ETSSSS--------RPPTQSIPK-------PVLTDEVSDSDLISMWSEIDSMDVSSIDRESL-------R  108
WCLaV_2      67  DPKGP-----------AVESL------------PTEITEARIAAMWTEIDN----FDVSSLDQE---SLK  106
CCGaV        58  PKFATRDGD--DGQEPENKGDE---------ADSSNISDIQLAEMWQEIDL----MDVTSIDSE---SLK  109
LLV           2  ------------------------------------TSALSVYTMISAADT----TGVEEFS-------T   24
RSV          17  ----------------------------------NDISKDALSYLTAHKA------DVVTFA-------G   39
RGSV         15  ----------------------------------KEWSDLLSEIFSKIR------ASIDGFANATADLAA   44
MSV          17  ----------------------------------NDISKDALKYLTDNKA------SVTTFH-------D   39
RHBV         13  ----------------------------------ERVTTLALKYITDHKD------TLVTFV-------G   35
EHBV         11  ----------------------------------ERVTALALKYIADHKD------TLVTFV-------G   33
UHBV         13  ----------------------------------ERVTALALKYITDHKD------TLVTFV-------G   35
IWSV         13  ----------------------------------ERVSTLALKYITEHKD------ILVAFA-------G   35
RVFV          2  ----------------------------------DNYQELAIQFAAQAVDR----NEIEQWV-------R   26
SFTSV         3  -----------------------------------EWSRIAVEFGEQQLNL----TELEDFA-------R   26
SFNV          4  ----------------------------------ENYRDIALAFLDESADS----GTINAWV-------N   28
UUKV          4  ---------------------------------PENWVRFAIEISDAQWEE----EEIREFI-------N   29
TOSV          4  ----------------------------------ENYRDIALAFLDESADS----GTINAWV-------N   28
Consensus_ss:    h              hhhhhhh                 hhhhhhhhhhhhhhhhh    hhhhhhh        h

                    *                                     *             *
CiVA        130  VFDYQGFNPDEVLRSLIINQRKNKVSAADFKSDILLMCSLAIIKGSI-----NEHNFKKLS--TEGQSTV  192
WCLaV_1     109  IFEYQGFNPDEILKSLIIAQRKNKISGDQFKTDILTLCAISIIKGSI-----NDHNFGKIS--KEGQDAI  171
WCLaV_2     107  IFEYQGFNPKAILISLMKSCDKNKIPKEQFKSDIITLCAISIIKGSI-----NSNNIKKVS--EEGQQEI  169
CCGaV       110  VFDHQGFKPNEILKSLMVQARKNKVSKEDFKTDILMMCAISIIKGSI-----NEHNFKKLS--TEGQTEV  172
LLV          25  LFAYEGFNPEMIHSHFAKIMTEKGIGEMEFVNDMRALITLGAMKGNY-----TMKNAGKIS--EAGRTKA   87
RSV          40  QIEYAGYDAATLIGILKDKG------GDTLAKDMTMCITMRYVRGTG-----FVRDVTKKVKVAAGSTEA   98
RGSV         45  GLEYQAFNPEKILRKLIASS----TSLDDFVKDMRDLLVARYTRGTSFLFNAKNSIEKAKD--KKKAEAI  108
MSV          40  QIGYAGYDAATLIGILKDKG------GATLAQDVVKMIVMRYVRGTG-----FVKDVTKKTKATAGSEEA   98
RHBV         36  QIEYNGYDAGKLLQILKRKA-----EGRDFGKDLCYLLVMRYTRGTG-----FVRDVRKKIKTAAGGDTA   95
EHBV         34  QIEYNGYDAGKLLQILKKKA-----EGRDFGKDLGFLLVVRYTRGTG-----FVRDVRKKIKTAAGGDTA   93
UHBV         36  QIEYNGYDAGKLLQILKKKA-----EGRDFGKDLCYLLVMRYTRGTG-----FVRDVRKKIKTAAGGDTA   95
IWSV         36  QIDYNGYDAGKLLKILQDKS-----KNRDFGKDLCHLLVMRYTRGTG-----FVRDVRKKIKVAAGGETS   95
RVFV         27  EFAYQGFDARRVIELLKQYG------GADWEKDAKKMIVLALTRGNK-----PRRMMMKMS--KEGKATV   83
SFTSV        27  ELAYEGLDPALIIKKLKET------GGDDWVKDTKFIIVFALTRGNK-----IVKASGKMS--NSGSKRL   83
SFNV         29  EFAYQGFDPKRIVQLVKERGT---AKGRDWKKDVKMMIVLNLVRGNK-----PEAMMKKMS--EKGASIV   88
UUKV         30  LFQYQGFDAAVVLSRIFELAKKADLSRDQMLRDIRALITLHLTRGNK-----LSSIEKRLS--EEGKKEF   92
TOSV         29  EFAYQGFDPKRIVQLVKERGT---AKGRDWKKDVKMMIVLNLVRGNK-----PEAMMKKMS--EKGASIV   88
Consensus_ss:    hhhh    hhhhhhhhhhhh     hhhhhhhhhhhhhhhhh              hhhhhhhh   hhhhhhh
```

```
                                              *
CiVA            193  IRLEKTYGIKRGSGRSEPANVVTISRIGATFPGKIIQLLVE---NKASSRKFMGP--LKSHTLPQFIR--  255
WCLaV_1         172  VKLENSYGIKRGSGRGMNSTVVTVSRIAATFPGIVIQLLRD---NKVSARKFIGP--LKSQTLPAVMR--  234
WCLaV_2         170  SRLETLYNIKRGGGRKERPDVITVSRIGATFPGKIVQLIHA---GKVQSRAFMGP--FSSSSLPPVMR--  232
CCGaV           173  QRLEKTYGIKRGSGRNEPANVVTISRIGATFPGKIIQLFLA---GKVTARKFIGP--FKSHTLPQYVR--  235
LLV              88  DGLYKKYNMKQGSLG-GDKKAVILPRVLSAFPELTTKVVLR--TPPRDFGTRTT-------RLPKYIK--  145
RSV              99  STLVSRYGIVSSVG--TNANAITLGRLAQLFPNVSHEVVRQIS-GVK---MAVDSSDLGLTGCDNLLWDY  162
RGSV            109  QVLINRYGVKKNAGD-NAVDQATLGRISQVLAYMALRVALQITDYHKPIIPLRP---ISTVDIKNAIIDV  174
MSV              99  AALVARYGLVSSVG--SNANAITLGRVAQLFPNVSFEVTKQFT-GLK---MAIDSSDLSMSGTDSLLWDF  162
RHBV             96  HEIVTHYGVVQSVG--DNADAITLGRLASLFPAVSMNIVRNVSTGAK---LAVDSSDLGSSGLDLLLWDF  160
EHBV             94  HEIVTHYGVVQSVG--DNADAITLGRLASLFPAVSMSIVKNVSSGAK---LAVDSSDLGTSGLDILLWDF  158
UHBV             96  HEIVTHYGVVHSVG--DNADAITLGRLASLFPAVSMNIVRNVSTGAK---LAVDSSDLGSSGLDLLLWDF  160
IWSV             96  HEIVTHYGIVQSVG--DNADAITLGRLAALFPNVSMTIVKSVSTGAK---LAIDSADMGTSGIDILLWDF  160
RVFV             84  EALINKYKLKEGNP---SRDELTLSRVAAALAGWTCQALVVLSEWLPVTGTTMDG---LSPAYPRHMM--  145
SFTSV            84  MALQEKYGLVERAE---TRLSITPVRVAQSLPTWTCAAAAALKEYLPVGPAVMNL---KVENYPPEMM--  145
SFNV             89  ANLISVYQLKEGNP---GRDTITLSRVSAAFVPWTVQALRVLSESLPVSGTTMDA--IAGVTYPRAMM--  151
UUKV             93  ALLKARYQLVDKAK---EAADLTLSRIAIANAGLTCRILPQVVAHTAVTRSRMES---LSADYPVCMM--  154
TOSV             89  ANLISVYQLKEGNP---GRDTITLSRVSAAFVPWTVQALRVLSESLPVSGTTMDA--IAGVTYPRAMM--  151
Consensus_ss:        hhhhhhh            hhhhhhhhhhhhhhhhhhhhhhh                     hhhh


                         *
CiVA            256  HQGFAAVIPTTLA----ESTRMFLLDIVTAYSVDQSISISPNKK-------NKPDLASLYEKQRSFI-QT  313
WCLaV_1_        235  HQAFAAVIPTALR----EKTRNFLLILITAFSVDQSINISPNKK-------EKPDVEGLFKLQENFIQVS  293
WCLaV_2         233  HQAFAAVIPRSMN----HKIKEFLLQLITAFSVDQSISINPNKK-------EKMDPVTTFSTQHNYIQVI  291
CCGaV           236  HQAFSAVIPSSLN----ERTKMYLLDIITAFSVDQSICISPNKK-------EKPDLSNLFEKQRAFV-QT  293
LLV             146  NPVFPSLVPKTLQ----GDVAKTFLWLYTVYSAEQSLVIS-----------QEKDFNAAFTAQKQFVTIA  200
RSV             163  VPQYIKLESETAPYCSTHSLSHILFVVHIIHSFQITKKTMPEGKKKERGLTKDIDMMKYTT---GLLVIT  229
RGSV            175  VPQFLYLKADQLDSK--TNSEAALYVIHLCYQVCVSERIMTKAQKDKHSVHTKSAMITHCM---GFVNLA  239
MSV             163  VPQYITLDSSSAPYCTTKSVAHILFSIHVIHAFLVTKKTMPEAKKKERGLLKDIDIIKYTT---GLLVIT  229
RHBV            161  VPQFITLDSLTAPYCTGKGMNTILFSLHVLHGALTTKKTMPDQKKKARSLVTDFELVKYTT---ELLVIT  227
EHBV            159  VPQFITLDSVTAPYCTGKGMNVILFSLHVLHGALTTRKTMPDLKKKARSLVTDFELVKYTT---ELLVIT  225
UHBV            161  VPQFITLDSVTAPYCSGKGMNIILFSLHVLHGALTTKKTMPDQKKKARNLVTDFELVKYTT---ELLVIT  227
IWSV            161  VPQFISLDSETAPFCNKKNANNVLLSLQILQGALTTKKTMPDQKKKLRNLITDYDFVKYTS---ELMVIT  227
RVFV            146  HPSFAGMVDPSLP----GDYLRAILDAHSLYLLQFSRVINPNLRG-----RTKEEVAATFT---QPMNAA  203
SFTSV           146  CMAFGSLIPTAGVS---EATTKTLMEAYSLWQDAFTKTINVKMRG-----ASKTEVYNSFR---DPLHAA  204
SFNV            152  HPSFAGIIDLDLP----NGAGATIADAHGLFMIEFSKTINPSLRT-----KQANEVAATFE---KPNMAA  209
UUKV            155  HNAFAGLIDETLP----EDSIKALVDAHRLYLLEFSRTINVKHRG-----MEAKEILDAND---SALQAG  212
TOSV            152  HPSFAGIIDLDLP----NGAGATIADAHGLFMIEFSKTINPSLRT-----KQANEVAATFE---KPNMAA  209
Consensus_ss:           h             hhhhhhhhhhhhhhhhhhhhh   hh          hhhhhhhhh   hhhhhh


CiVA            314  TADNKYPSEIVRK----------------------KIFTTI-----------------MID------YDS  338
WCLaV_1_        294  ADNIYPPESIRVK----------------------IFKSV------------------ILN------YDD  317
WCLaV_2         292  HDGEYPPENIKKQ----------------------IFKTIS-----------------INF------DDL  316
CCGaV           294  TSDAEYPPEIVRK----------------------KIFSTI-----------------LLD------YDQ  318
LLV             201  FNSSVPDETTRIG----------------------MFKAKLED------------LVQAISDFKDFTDGE  236
RSV             230  CKSKNLSDKKKEEGRKKVLDEFITNG----KVKTTIFDALA-----------------GMS------VNT  272
RGSV            240  MDNSSVVSDDKIA----------------------GRRMISGPWGLQETALDATGCACIIDVVDFCCRGH  287
MSV             230  CQSKNLNEAKKKSGRTKVCEPYCVNE----KFKESFLALLA-----------------SFG------KNV  272
RHBV            228  CNAKNLKVEKKTIYRESLMRPFQEGAGDALGFKETFWTSLA-----------------KVS------TGC  274
EHBV            226  CNAKNLKAERKTAYRKSLMSSFQEGAGGDLGFKESFWTSLA-----------------KVS------TGC  272
UHBV            228  CNAKNLKAEKKTSYRESLMRPFQEGAGDALGFKETFWTSLA-----------------KVS------NGC  274
IWSV            228  CSAKNLTDEKKQTYRRKLVSAFQTDD---SGYKQEFWDALG-----------------QVS------VGC  271
RVFV            204  VNSNFISHEKRRE----------------------FLKAFG-----------------LVD------SNG  228
SFTSV           205  VNSVFFPNDVRVK----------------------WLKAKG-----------------ILG------PDG  229
SFNV            210  MSGRFFTREDKKK----------------------LLIAVG-----------------IID------EDL  234
UUKV            213  LASSFLTPSQKRA----------------------YLLSFK-----------------LVD------GNG  237
TOSV            210  MSGRFFTREDKKK----------------------LLIAVG-----------------IID------EDL  234
Consensus_ss:        h      hhhhhh                      hhhhhh
```

```
CiVA           339  L----------LTTARALLKVIPDFTIVTKENFVSDINSTHI------------------  370
WCLaV_1_       318  L----------LTCARSISKFINDIIIPTRDEFMSDISQL--------------------  347
WCLaV_2        317  L----------VTAR-KIVALTGNLEIPSKDEFNSAVMSFN-------------------  346
CCGaV          319  L----------LSTARAILKVFPEFVIVSKENFNADINATHI------------------  350
LLV            237  V----------PTN--RSDA----------RVARDAITTL--------------------  254
RSV            273  ISTYGNQTRLYLAQQSKLMKILAENTSKTATEVSGLVKEFFEDEAEGADD----------  322
RGSV           288  K----------VTDAVAPV-----------RLFRLAIECI-KDTADLKDAGVKLKTLVDK  325
MSV            273  VCSYGTQVKQFLAEQCSLMKTIVDNSSKTQDEMKALIIEFFEEE----------------  316
RHBV           275  VRKMKKDAQAYLSERTPLLKVFVENCNKSEDEVAEAIKSYFVTGA---------------  319
EHBV           273  VKKMKKDAQAYLSERTSLLKLFVENCNKTEAEVTEAIKSYFVTDA---------------  317
UHBV           275  VRKMKKDAQTYLSERTSLLKVFVDNCNKAEAEVTEAIKAYFVTES---------------  319
IWSV           272  VRKLKKDAQNYLKDRTTVLKVMVENCTKTEAEAIEAIKAFFAPEDV--------------  317
RVFV           229  K----------PSAAVMAAA----------QAYKTAA-----------------------  245
SFTSV          230  V----------PSRAAEVAA----------AAYRNL------------------------  245
SFNV           235  V----------LASAVVRSA----------EKYRAKVGK---------------------  253
UUKV           238  K----------VNKAVQQAA----------TVLRSLI-----------------------  254
TOSV           235  V----------LASAVVRSA----------EKYRAKVGK---------------------  253
Consensus_ss:                   hhhhhhhh          hhhhhhhh
```

Figure S3. Multiple alignment with PROMALS3D of the putative nucleocapsid protein (NP) encoded by citrus virus A (CiVA) and homologous proteins of representative phleboviruses, tenuiviruses and the phenui-like viruses more closely related with CiVA. Amino acids that fold in strands and helices and are in blue and red, respectively. Consensus secondary structure elements, predicted by PROMALS3D, are reported at the bottom, with the strands and helices indicated by e and h, respectively (in green are indicated the helices conserved only in the NP of CiVA, CCGaV, WCLaV-1 and WCLaV-2; in bold are two α-helices conserved in all viruses and shown to be essential for the NP protein oligomerizarion in RVFV, UUKV, RSV (Katz et al., 2010, Ferron et al. 2011, Lu et al., 2017). Blue asterisk (*) indicates a tyrosine involved in RNA binding in RVFV and UUKM (Raymond et al., 2012; Mottram et al., 2017), while green asterisks mark the three positive charged amino acids predicted to be involved in the protein RNA binding activity in RVFV and RSV (Ferron et al., 2011; Lu et al., 2017). The pink asterisk denotes the phenylalanine residue shown to have an essential role for infectivity in RFVF (Mottram et al., 2017).

Table S1. Primers used in this study

Name	Strand	Sequence (5' to 3')	Genomic RNA	Position	Length amplicon (nt)	Used for detection
Ka-1	vc	TCCTGATGAAGTCTTAAGATCGC	RNA2	2235-2257	620	RT-PCR
Ka-3	v	TTGCAGTAGTGAGAAGGGAGT	RNA2	1638-1658		
Ka-32	vc	CCTTCAAATATTAAGAGCTGAGCT	RNA1	274-297	297	5'RACE
Ka-33	vc	GGATCAACATGTTGATAATTTAACC	RNA1	249-273	273	5'RACE
Ka-18	v	CTAGGCCATATCTAGAACTAAGC	RNA1	140-162	1193	RT-PCR
Ka-16	vc	GAGTGAGGGCTTCTCTAGCAA	RNA1	1312-1332		
Ka-15	v	CAATAACGAAATCAGGATCCCAG	RNA1	1286-1308	1379	RT-PCR
Ka-14	vc	TTGGTCAGAAGCTAAGTTTGCA	RNA1	2643-2664		
Ka-5	v	CTGATGCCGACATTAACCTAGCT	RNA1	2561-2583	1033	RT-PCR
Ka-12	vc	GGTATCTTCCAGCAAGCTCAAA	RNA1	3572-3593		
Ka-13	v	CTATTAGGTCCAGGCAGCCTAT	RNA1	3257-3278	1057	RT-PCR
Ka-10	vc	GATCTAGCCACTCTGAAAGTG	RNA1	4293-4313		
Ka-11	v	GAGAGTCATCATAGGATCTAGAT	RNA1	4268-4290	1217	RT-PCR
Ka-8	vc	GAAGAGCTTCCATCCTATACTAAC	RNA1	5461-5484		
Ka-9	v	GTTCATTGAAAGAGCAAACATCTG	RNA1	5036-5059	1550	RT-PCR
Ka-6	vc	CAAGATAGAGCCAACTCTGTAC	RNA1	6564-6585		
Ka-29	v	AACAACATCACACTGCTGACTA	RNA1	6412-6433	279	3' RACE
Ka-30	v	CATCTAACTCTCTCTCTCTGCCA	RNA1	6470-6492	222	3' RACE
Ka-36	vc	TGTTAGGGAGGTCGAACTTGTA	RNA2	225-246		5'RACE
Ka-35	vc	TCCTTTGCCAGATCATCAAACC	RNA2	256-277		5'RACE
Ka-34	vc	TACTGGAATCTTGTTCCTTCACTA	RNA2	281-304		5'RACE
Ka-20	v	TTAGCAGAGCTGGGAAAAATG	RNA2	160-180	843	RT-PCR
Ka-21	vc	TAGCTGAAATGGAAGACTTTGCA	RNA2	980-1002		
Ka-40	v	GCCTTACCACCTGCAGTTCTT	RNA2	819-839	898	RT-PCR
Ka-41	vc	AAGTACCCTTCAGAGATTGTGAG	RNA2	1694-1716		
Ka-24	v	GTCACGATTGTAAAATCAGGAAT	RNA2	1598-1620	998	RT-PCR
Ka-25	vc	GAAGAGCATTATCGTGAGTTCC	RNA2	2574-2595		
Ka-38	v	GCCTGCTCGAAACTCTCAAGA	RNA2	2474-2494	267	3' RACE

Table S2 Contigs coding for peptides with significant amino acid (aa) sequence identity with proteins of CCGaV.

Protein	Contigs ID	Length (nt)	aa-Identity (%)	aa -position*
RdRp	NODE_17015	47	93	19-33
	NODE_7207	50	81	184-199
	NODE_20526	65	79	381-399
	NODE_4473	53	100	527-543
	NODE_20299	56	89	776-793
	NODE_27930	47	80	1013-1027
	NODE_5515	60	100	1117-1135
	NODE_24514	49	94	1182-1197
	NODE_2299	68	100	1224-1235
	NODE_15151	57	79	1353-1369
	NODE_3044	66	89	1769-1786
NP	NODE_3249	172	38	26-57
	NODE_5667	79	55	83-102
	NODE_1501	61	61	87-104
	NODE_5489	38	73	109-119
	NODE_3098	127	63	116-155
	NODE_8024	106	96	178-203
	NODE_2373	58	78	219-236
	NODE_2080	89	62	233-261
	NODE_1563	72	74	257-279
	NODE_2838	67	65	289-305
	NODE_6834	46	79	303-316
	NODE_298	80	73	319-340
MP	NODE_2570	101	59	38-64
	NODE_3279	59	89	62-79
	NODE_6661	50	93	85-99
	NODE_2830	84	96	124-146
	NODE_527	39	100	151-162
	NODE_1449	116	72	157-192
	NODE_2180	104	86	190-218
	NODE_2282	72	80	223-242
	NODE_3988	48	77	236-248
	NODE_1231	74	96	249-271
	NODE_2374	70	82	268-289
	NODE_2363_	94	77	295-324

* aa position with respect to CCGaV proteins RdRp (YP_009422199), NP (YP_009407931) and MP (YP_009407930)

Table S3. Amino acid sequences identities between CiVA and other related (-ss) RNA viruses

Virus	BlastP results						Pairwise identity (%)			
	RdRp		MP		NP		RdRp	RdRp core	MP	NP
	E value	Identities	E value	Identities	E value	Identities				
CCGaV	0.0	1674/2184 (77%)	0.0	267/409 (65%)	4,00E-160	217/365(59%)	78.48	84.11	67.18	60.64
WCLaV-1	0.0	1376/2176 (63%)	2,00E-134	204/396 (52%)	3,00E-121	178/352 (51%)	63.10	72.85	52.09	50.72
WCLaV-2	0.0	1262/218 (58%)	6,00E-116	191/405 (47%)	4,00E-95	139/265 (52%)	57.67	74.05	47.44	44.71
LLV	0.0	713/2151 (33%)	1,00E-33	97/321 (30%)*	4,00E-12	55/220 (25%)	32.54	45.59	28.12*	23.98

* The function of this LLV protein (ID:AUW34409.1), encoded in the RNA2, is unknown
